# Managing Patient and Clinician Expectations of Phage Therapy in the United Kingdom

**DOI:** 10.3390/antibiotics12030502

**Published:** 2023-03-02

**Authors:** Joshua D. Jones, Helen J. Stacey, Arlene Brailey, Mehrunisha Suleman, Ross J. Langley

**Affiliations:** 1 Clinical Microbiology, Ninewells Hospital, NHS Tayside, Dundee DD2 1SG, UK; 2 Infection Medicine, Edinburgh Medical School: Biomedical Sciences, University of Edinburgh, Chancellor’s Building, 49 Little France Crescent, Edinburgh EH16 4SB, UK; 3 Public Health, Kings Cross Hospital, Clepington Road, Dundee DD3 8EA, UK; 4 Antibiotic Research UK, Genesis 5, York Science Park, Church Lane, Heslington, York YO10 5DQ, UK; 5 The Ethox Centre, University of Oxford, Li Ka Shing Centre for Health Information and Discovery, Old Road Campus, Oxford OX3 7LF, UK; 6 Department of Paediatric Respiratory and Sleep Medicine, Royal Hospital for Children, 1345 Govan Road, Glasgow G51 4TF, UK; 7 School of Medicine, Dentistry & Nursing, University of Glasgow, Glasgow G12 8QQ, UK

**Keywords:** bacteriophage, phage therapy, patient, expectation, ethics, United Kingdom

## Abstract

Bacteriophage (phage) therapy is a promising alternative antimicrobial approach which has the potential to transform the way we treat bacterial infections. Phage therapy is currently being used on a compassionate basis in multiple countries. Therefore, if a patient has an antibiotic refractory infection, they may expect their clinician to consider and access phage therapy with the hope of improvement. The expectations of clinicians may be similar and may also include expectations around data collection. However, there are multiple biological and practical barriers to fulfilling patient and clinician expectations. While it is possible to access phage therapy, the path to acquisition is not straightforward and expectations therefore need to be managed appropriately to avoid raising false hope and undermining confidence in phage therapy. Phage scientists have an important contribution to make in educating clinicians and the broader public about phage therapy. However, it is clinicians that are responsible for managing the expectations of their patients and this relies on clear communication about the barriers and limitations.

## 1. Background

Bacteriophages (phages) are naturally occurring viruses that generally infect bacteria in a species-, and sometimes even strain-, specific manner. Collectively phages are the most abundant “living” entity on the planet and can be found wherever bacteria are found. Independently discovered in 1915 and 1917, phages were first used to treat bacterial infection in 1919, and the use of phages to treat bacterial infection became known as phage therapy [1]. Thereafter, phages experienced a “golden age” and were used widely. Due to a variety of factors, not least the introduction of antibiotics, the use of phages declined sharply in the geopolitical West. Meanwhile, phage therapy remained in widespread use in the geopolitical East, where it remains a valuable antimicrobial tool [2]. Today, the antimicrobial resistance crisis has rekindled interest in phage therapy globally, with phages currently being used, albeit sporadically, on a compassionate use basis across Europe, Australia and the US [3,4,5].

Phages have also been used to treat patients in the United Kingdom’s National Health Service (NHS) and it is from that perspective that this article is written. The NHS is divided into geographically distinct authorities referred to by different terms depending on where they are in the UK. For the purposes of this article, all NHS authorities will be referred to as Trusts. So far in the UK two cystic fibrosis patients at Great Ormond Street Hospital (London, UK) and ten diabetic foot infection patients in two Scottish hospitals have received phage therapy [6] (Young and colleagues, in submission, 2023). Recently, Health Improvement Scotland’s Health Technologies Group have recommended that phage therapy be considered for difficult-to-treat infections [7]. Naturally occurring phages (i.e., not classed as genetically modified) are classified by the UK regulator (the Medicines and Healthcare Products Regulatory Agency [MHRA]) as a biological medicine and may be used on an unlicensed basis (known as a “special”) when a clinician determines that their patient’s clinical needs cannot be met by licensed alternatives and according to MHRA guidance [8]. Patients for whom unlicensed phage therapy may be appropriate include those with: wholly antibiotic-resistant infections; antibiotic-susceptible but clinically recalcitrant chronic infections; reasonably foreseen acute risk to life or limb despite appropriate antibiotic treatment; other patient-specific factors that preclude the use of appropriate antibiotics (e.g., renal failure, allergy, drug–drug interactions or intolerable side effects) or cases where further medical intervention is preferred to surgery (e.g., high-risk surgical candidate).

The use of phage therapy as an unlicensed medicine is underpinned by a sizeable body of reassuring evidence about the safety and efficacy of phage therapy. For example, there have been 13 safety or clinical trials of phages since the year 2000, all of which have shown phages to be safe by a variety of routes of administration [9]. Meanwhile, there have been >2200 clinical reports of phage since the year 2000, among which 79% of 1904 patients showed clinical improvement and 87% of 1461 patients achieved bacterial eradication; many of these cases were refractory to antibiotic therapy [10]. Clinical trials meanwhile have not consistently demonstrated efficacy, although this is considered to arise from shortcomings in the trials themselves and not to reflect the mechanistic ability of lytic phages to infect and kill their bacterial hosts [9].

Phage therapy is a broadly applicable antimicrobial strategy that has the potential to transform the way we treat bacterial infections. Additional advantages of phage therapy include that some phages possess enzymes capable of degrading biofilms, the polysaccharide matrices considered to play a key role in many chronic infections, such as those caused by *Pseudomonas aeruginosa* [11]. Moreover, as phages are not human pathogens, they are suitable for use in patients with immunodeficiency [12]. Notably, unlike many antibiotics, such is the specificity of phages that the commensal flora is left largely intact [13], reducing adverse effects associated with the loss of commensal organisms, including the acquisition of opportunistic pathogens. Intriguingly, phages may also act synergistically with antibiotics and there is some evidence that adjunctive phage therapy can “re-sensitise” bacteria to antibiotics [14]. While preformulated phage cocktails may be able to meet most clinical needs, a library of phages can be used to devise personalised phage formulations. Although phages are unlikely to completely replace conventional antibiotics, except in limited clinical circumstances, phage therapy nonetheless has the potential to be as radically transformational to medicine as antibiotics once were.

## 2. Setting the Context: Patient and Clinician Expectations of Phage Therapy

Patients with antibiotic-refractory infections are similar to individuals suffering from an “orphan” (rare) disease and face similar challenges, such as the availability of suitable treatments [15]. Box 1 illustrates a typical patient story. Understandably, some patients explore alternative therapies and may read about the potential of phage therapy. Much of the evidence around the safety and efficacy of phage therapy comes from individual clinical case reports, which patients may find easier to understand compared with clinical trial data. For patients, reading about phage therapy, particularly clinical case data, will create a variety of expectations including a good safety profile and potentially permanent resolution of their infection. Patients may also expect that their clinician will have an awareness or knowledge of phage therapy and consider phage therapy to be a safe adjunct to conventional antibiotics. Knowledge about phage therapy amongst UK clinical teams is limited, and historically they have taken a cautious approach, however, their expectations may be similar to that of their patients; first, that they can access phage, second that phage therapy might help and lastly that it will be safe. Additionally, clinicians using phage therapy may also expect to be able to collect and publish data about their use of phage therapy.

Box 1 **Caroline’s story. Written by Arlene Bailey in January 2023, with Caroline’s consent.**

**Caroline’s story**

Caroline, an English tutor, has been virtually housebound for the last 6 years due to a recurring multi drug resistant urinary tract infection, acquired after a simple gynaecological procedure. Referral to three different urologists, multiple short and longer term courses of 11–12 different antibiotics over 6 years, and even gentamicin bladder installations, have all failed to eradicate the causative bacteria.

Infection has devastated Caroline’s life, costing her the job she loved, hobbies, family and social life. It has impacted everyone and shaken her mental health. She now spends her days trying to distract herself from the symptoms and “killing time” while her husband has become her carer.

Side effects of numerous antibiotics have also taken their toll on her gastrointestinal system, and Caroline can no longer tolerate any oral antibiotic treatment. With her General Practitioner at a loss to know what else to do, and no practical help offered by urologists, Caroline has sought other treatment options. Finding others who successfully used bacteriophage treatment, she booked an appointment at the bacteriophage centre in Georgia in 2020. Caroline initially tried phage treatment remotely but encountered several practical and clinical difficulties which led to the decision to go to Georgia in person. The war with Russia put this idea on hold indefinitely.

Caroline says “I feel angry that initial urine testing and antibiotic treatment was not adequate enough to accurately identify and properly treat this infection, and I am paying the price now. We desperately need other therapies like phage treatment for those of us for whom traditional antibiotic treatment has failed. I just want my life back.”

## 3. Barriers to Success

While it is possible to use phage therapy in the UK, it is currently not straightforward. The process initially involves the patient’s clinician deciding whether phage, as an unlicensed medicine, may be appropriate. If phage therapy is to be considered, then there are biological and practical barriers to successful phage therapy (Table 1). It may be that no suitable phage(s) have been identified for a patient; although it is likely that suitable phage(s) will exist in nature. Although the definition of phage suitability can be multifaceted [16], for the purposes of this manuscript “suitable” phages are simply defined as those able to infect and kill the target bacterial species. If suitable phages are identified, access may be limited. The “manufacturing quality” of potential phages may also be a barrier. Similarly, the lack of understanding and established infrastructure within the healthcare systems charged with assessing the quality and efficacy of unlicensed medicines for clinical use may represent a further barrier.

Although all NHS Trusts have unlicensed medicines policies, and thus would be able to use phage as an unlicensed medicine, the ease with which this may be achieved will vary widely between Trusts and is far from equitable. Staffing and clinical pressures may limit the consideration of phage. Likewise, unfamiliarity regarding handling, storing or administering a live “virus” may also prove problematic. Like all novel medications, there may be a degree of scepticism which may also prove a substantial barrier to overcome.

Currently, therapeutic phages in the UK are imported and the logistics can be challenging. Importation itself can only be undertaken by an appropriately licensed organisation [8]. There are also cost implications, with funding needed to cover the costs of sending clinical isolates to phage labs and the subsequent importation of any phages; some phage sources may also require a financial contribution. NHS Trusts not appropriately licensed for importation will face additional barriers as private importers of unlicensed medicines may require in-depth information about the phage from the source, potentially requiring formal agreements to cover information transfer.

Once appropriate phages have been sourced, successful administration to a patient is the next potential barrier to success. At the time of writing, only three NHS Trusts have used phage therapy, meaning there is scant experience of phage administration in the NHS. Decisions about treatment plans (such as dosage, route and duration) may be challenging for inexperienced clinical teams but should be evidence-based wherever possible and may be guided by the existing clinical phage literature or written advice from established international clinical phage centres. We note that no specialist clinical expertise is required for the practical administration of phage suspensions and all Trusts possess the necessary clinical skills for phage administration. Therefore, patients should only be treated with phage in the NHS Trust in which they would already be receiving care and all Trusts should be able to independently consider treatment plans for phage therapy [17]. Crucially, this will stimulate the valuable development of distributed expertise across the UK, rather than the inappropriate creation of specialty-specific centres and is in line with conceptually considering phages as we would a new antibiotic (i.e., all Trusts would be expected to use a new antibiotic independently and not refer to other Trusts). Safety is the key expectation for both patient and clinician. Whilst available trial and clinical evidence suggest that phage therapy is safe [9,10], the use of impure phage preparations would represent a safety concern as impure phage preparations containing high levels of bacterial toxins and/or immunostimulatory molecules could cause adverse clinical reactions [18,19].

Following administration, the patient and clinician would expect treatment efficacy. A successful outcome depends on the results of pre-treatment laboratory phage sensitivity testing. However, even if the patient’s bacteria were shown to be susceptible to phage in vitro, this does not guarantee treatment success. Treatment failure may be caused by bacterial resistance to the phage(s) during treatment and alternative phages may not be available [20]. Similarly, heterogeneity within the infecting bacterial community may further complicate outcomes since some variants within that population may be more likely to have or develop resistance to phage [21]. Likewise, treatment failure may also occur when phages are used over a long period of time, which may result in a host response and rapid neutralisation of phage [22]. Even if the target pathogen is eradicated from a site of infection, overgrowth by other pathogens or opportunistic organisms could theoretically maintain an infection. The timing of phage administration during an infection will also influence the outcome. Phage must be given sufficient time to kill the bacteria. The timing of phage administration within the clinical course of infection also requires consideration, with late administration risking the infection being already irretrievable.

Successful phage therapy depends on the phages encountering the target bacteria in sufficient numbers to sustain phage replication [23]. To encounter the bacteria the phages must therefore be administered to the site of infection. In some specific cases, such as chronic urinary tract infections, small populations of bacteria may remain inaccessible to phages and potentially contribute to treatment failure [24]. Intracellular bacteria pose a particular challenge to phage therapy. While there is evidence that some phages can penetrate eukaryotic cells, the therapeutic significance remains unclear [25,26]. Intracellular bacteria may therefore be protected from phages, for example in mycobacterial infections [6,27,28]. Whilst phage therapy may be able to resolve acute episodes of mycobacterial infection, when many bacteria are extracellular, subsequent treatments may be required if the infection recurs [29]. The potential need for further courses months or even years later is a barrier to continued treatment success as access to repeated courses of phage may be limited. The need for repeated courses of phage is also a barrier to patients who have an underlying predisposition to infection. In these cases, phage therapy may be useful in resolving a distinct infectious episode, but phage therapy cannot address any underlying causes of infection. For example, recurrent urinary tract infections may be caused by anatomical predispositions or indwelling devices. At this early stage in the development of phage therapy infrastructure, it may not be possible to support patients with recurrent infections because of the limited capacity to source new phages each time. Continued access to phage therapy may also be a barrier if a patient’s clinical care team changes, for example, if the patient relocates into another Trust or transitions from paediatric to adult care within the same Trust [29]. However, access to repeated courses of phage therapy will be a surmountable problem once sustainable and scalable phage infrastructure is in place.

## 4. Managing Patient and Clinician Expectations of Phage Therapy

To ensure safe and sustained access to phage therapy in routine clinical practice it is essential that patient and clinician expectations are effectively managed. Failure to manage expectations by not providing information about the barriers to success may lead to false hope and loss of confidence amongst clinicians and patients alike. Moreover, in the event of treatment failure, clinicians may consider that phage therapy simply does not work. This could lead to a scenario analogous to that experienced to some extent in the 1920s and 1930s when enthusiasm about phages led to the injudicious use of phage without testing to see if a patient’s bacteria could be killed by the proposed phage [30]. This inevitably led to treatment failures and helped undermine broader confidence in phage therapy. Such considerations point to the ethical need for managing expectations and mitigating false hope.

Clinicians are responsible for managing the expectations of their patients. Given the many barriers to success, each of which may prevent access to or the success of phage therapy it is important that, if phage is considered suitable, patients are not given false hope. Prior to considering phage as a potential treatment option for a patient, clinicians should be satisfied that licensed alternatives are not meeting a patient’s clinical needs, potentially involving input from appropriate clinical colleagues. For example, a case review by colleagues in medical microbiology or infectious diseases and/or multi-disciplinary team input or equivalent may be valuable and any agreed rationale for the use of an unlicensed medicine documented. Before discussing phage as a potential treatment option with a patient, clinicians should ideally have identified a likely source of phage and evaluated the feasibility of their Trust acquiring phage. If suitable phage(s) cannot be found and/or if phages cannot be accessed by their Trust within a clinically relevant timeframe, then discussions about phage with the patient would likely create unnecessary false hope. Patients may be disappointed if their consultant decides that phage therapy is not appropriate. Where phage therapy is not deemed appropriate the rationale behind the decision and an alternative treatment plan must be clearly communicated to the patient. If a patient disagrees strongly with the decision, they may seek a second opinion from an alternative consultant within their Trust. If a second opinion agreed with the first, then a patient would not be able to access phage therapy via the NHS and the focus should be on clearly explaining the rationale for the decision to the patient. If there is a reasonable chance that suitable phage(s) can be sourced and accessed in a timely fashion, then clinicians need to discuss the potential use of phages with the patient to enable them to provide informed consent for samples of the bacteria to be sent for phage sensitivity testing. The patient should leave this initial discussion aware of how a sample of their bacteria will be obtained and that phage sensitivity testing may show that their bacteria cannot be killed by the phage and is therefore not a guarantee of phage provision.

Clinicians may wish to collect data and should ensure that data collection may not be viewed by regulators as an unauthorised clinical trial [31]. Health Research Authority guidance provides a definition of research [32]. While it is appropriate that data from clinical applications of unlicensed phage therapy are shared, for example in published case reports, the data collected must be limited to that necessary for the care of the patient. Unlicensed phage use must be in response to genuine clinical needs arising and should not be seen as an alternative route to data collection about phage therapy. The collection of data beyond that required for the care of the patient risks becoming an unauthorised clinical trial, even if only one patient is treated [31]. Where clinicians or others involved in the oversight of unlicensed medicines require further clarification as to what is or is not research, Health Research Authority guidance should be consulted and if appropriate the matter brought to the attention of local research governance structures and, if needed, the MHRA [32].

Arguably, it is largely incumbent upon phage scientists to manage the expectations of clinicians, who are often enthusiastic about trying phages but have had limited exposure to the biological and practical complexities involved. Phage scientists can help manage clinician expectations by educating clinicians about phage therapy, although this must not stray into soliciting its use. Phage scientists can also play an important role in collating resources for clinicians, for example by undertaking systematic reviews. The phage community must also seek to explain the complexities of phage therapy by providing peer-reviewed literature aimed at clinicians. Phage scientists and clinicians will play different roles in managing patient expectations. Phage scientists may undertake public engagement activities to increase awareness of phage therapy among the public and get the message across that phages are “good viruses”. Public engagement will also play a key role in making the public, and potential patients, aware of the practical, biological and clinical challenges to successful phage therapy. However, to avoid potentially generating false hope, which clinicians may later have to let down, public engagement activities should present a realistic and balanced view of phage therapy.

## 5. Conclusions

Phage therapy is a promising antimicrobial strategy that has the potential to transform the way we treat bacterial infections. Patients and clinicians may understandably have high expectations of phage therapy as an alternative treatment for antibiotic refractory infections. Clear communication about the practical and biological barriers to accessing phages or treatment success will be important, especially at this early stage of clinical phage infrastructure. Phage scientists will play an important role in informing clinicians. Meanwhile, clinicians are responsible for managing the expectations of their patients and should be prepared for upfront discussions about barriers to success. Failure to manage these expectations could lead to false hope and undermine confidence in phage therapy. While we acknowledge the substantial potential of phage therapy, there is an ethical imperative to avoid false hope, particularly in the high-stakes antibiotic refractory cases currently most likely to be suitable for phage therapy.

## Figures and Tables

**Table 1:** Practical and biological barriers to successful phage therapy.

Practical Barriers to Success	Biological Barriers to Success
No suitable phage(s)	The bacteria are not susceptible to the phage(s) used
Suitable phages inaccessible	Bacterial resistance to phage(s) develops
Uncertainty about phage quality	Immune response against phage(s) reducing efficacy
Little or no knowledge about phage within the NHS (in general and around developing treatment plans)	Phages not used to target all the bacterial species responsible for the infection
Limited NHS capacity to handle complex unlicensed medicines requests	Phages applied too late to change the outcome of the infection
Funding for export of clinical isolate(s), access to phage(s), import of phage(s)	Reservoirs of bacteria may remain (e.g., intracellular or uropathogenic pathogens)
Identifying a suitably licenced importer	Repeated infections caused by an existing predisposition to infection not resolvable by phage therapy
Access to repeated courses of phage therapy	Impure phage preparations could elicit adverse effects (e.g., due to endotoxin)

## Data Availability

Data sharing is not applicable to this article as no datasets were generated or analysed during the current study.

## Abbreviations

MHRA	Medicines and Healthcare Products Regulatory Agency
NHS	National Health Service
